# Overcoming the Lignin Barrier in Brewer’s Spent Grain: Synergistic Effect of Additives on Enzymatic Hydrolysis

**DOI:** 10.1021/acsomega.5c04410

**Published:** 2025-11-17

**Authors:** Jozianny Bárbara de Almeida, Douglas Porto Vaz, Fernanda Ferreira Freitas, Gabriel Luis Castiglioni, Inti Doraci Cavalcanti Montano, Carlos Alberto Galeno Suarez

**Affiliations:** † Federal University of Goiás, Institute of Chemistry, Goiânia, Goiás 74690-900, Brazil; ‡ Federal University of Goiás, Agronomy School, Goiânia, Goiás 74605-220, Brazil

## Abstract

Brewer’s spent grain (BSG), an abundant lignocellulosic byproduct, has significant potential for biofuel and biochemical production, but its recalcitrant structure, particularly lignin, hinders enzymatic hydrolysis. This study evaluated the effect of additives on the enzymatic hydrolysis of Ca­(OH)_2_ pretreated brewery residue to improve cellulose accessibility and glucose recovery. Nine additives were initially tested, of which PEG 6000, Tween 80, and a cationic polymer improved hydrolysis yield. Response Surface Methodology (RSM) with Central Composite Design (CCD) was applied to optimize additive concentrations. The synergistic use of PEG 6000 and the cationic polymer achieved up to 87.9% glucose recovery with high model reliability (*R* ^2^ = 94%). The optimized conditions increased glucose release at different solid loadings, with improvements of 34.35%, 30.4%, and 15.7% for 5%, 10%, and 15% solids, respectively. While higher polymer concentrations showed some yeast inhibition, PEG 6000 exhibited no adverse fermentation effects. These findings demonstrate that the synergistic application of PEG 6000 and a cationic polymer effectively mitigates lignin barriers, increases the efficiency of enzymatic hydrolysis, and supports the sustainable valorization of BSG into fermentable sugars for bioeconomy applications.

## Graphical abstract

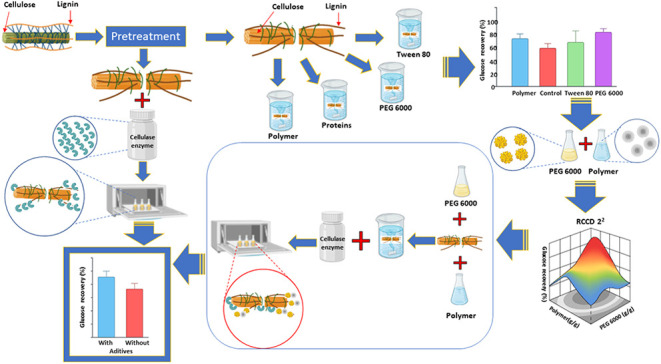

## Introduction

Lignocellulosic biomass is a plentiful renewable biomaterial that is often underutilized. It has the potential to be used in the sustainable production of biofuels and platform chemicals. One promising example is brewer’s spent grain, an agro-industrial byproduct that represents a poorly explored resource capable of yielding valuable biomolecules.^1^ This knowledge allows researchers to tailor their approaches to maximize the yield of desired products while minimizing waste and energy consumption. By understanding the complex structure of biomass, scientists can develop innovative strategies to break down its components into higher value-added products efficiently.^2,3^

The products that can be derived from this material include lactic acid, xylitol, prebiotic oligosaccharides, oleaginous yeast, and potential use for the production of biogas, ethanol, biobutanol, among others.^4–7^ These products have a wide range of commercial applications.^3,7^

However, brewer’s spent grain has a complex composition and recalcitrant structure, necessitating pretreatment methods to extract valuable components effectively.^8^ Different biomass sources exhibit varying chemical compositions, which require specific pretreatment methods and conditions to optimize extraction. These methods can involve physical, chemical, biological, or physicochemical degradation techniques. The challenge lies in finding cost-effective solutions and implementing environmentally friendly practices to promote sustainable development.^9,10^

One promising approach to overcoming these challenges is the use of alkaline pretreatment. Calcium hydroxide is considered a cheaper base compared to caustic soda (NaOH). Additionally, the lime left after pretreatment has no negative impact on the environment.^11^ Alkaline pretreatments are cost-effective and result in cellulose-rich biomass, leading to higher glucose yield.^12^ For this reason, the choice of alkaline pretreatment with calcium hydroxide, aligning with green technology principles that promote sustainable development and enhance the biodegradability of brewer’s spent grain.

On the other hand, pretreatment of biomass also produces various inhibitory compounds in the hydrolysates. These compounds lead to the formation of pseudolignin on the substrate surface, hindering enzymatic digestibility. Pseudolignin acts as a barrier, blocking cellulase access to cellulose.^13^ For this reason, an operational strategy must be employed to improve cellulose hydrolysis. One effective approach is to add compounds that have both hydrophobic and a hydrophilic component to the hydrolysis reaction mixture. For example, nonionic surfactants can be used to further reduce residual lignin interactions, thereby enhancing enzymatic access and improving overall hydrolysis efficiency.^14–16^

In the enzymatic hydrolysis of lignocellulosic material, additives such as Triton X-100 (Poly­(ethylene glycol) *p*-(1,1,3,3-tetramethylbutyl)­phenyl ether), Tween 20 (Polysorbate 20), Tween 80 (Polysorbate 80), poly­(ethylene glycol) (PEG), and bovine serum albumin protein (BSA) are commonly tested nonionic surfactants.^17,18^

In a study conducted with four different formulations of the surfactant Tween in the enzymatic hydrolysis of sugar cane bagasse, a significant increase in the hydrolysis yield rate was observed.^19^ In addition, new research demonstrated that the incorporation of PEG 4000 and Tween 80 in the enzymatic hydrolysis of bamboo led to a 35.2% increase in glucose yield.^20^ In addition, the addition of BSA in the enzymatic hydrolysis of sugar cane bagasse pretreated by steam explosion showed a positive effect, with a 20% increase in glucose yield.^21^

In the same context, the use of ionic Poly­(ethylenimine) (PEI) and Polyacrylamide (C-PAM) and nonionic Poly­(vinyl pyrrolidone) (PVP), Poly­(vinyl alcohol) (PVA), PEG water-soluble polymers were tested on substrates containing lignin (grasses) and without lignin (synthetic cellulose). In the test, it was found that the addition of the polymers led to an increase in hydrolysis yield, with the increase with nonionic polymers was significantly higher when compared to the use of cationic polymers.^22^

In contrast to previous studies that evaluated hydrolysis conditions at low solids loads and without considering the subsequent fermentation step, this study emphasizes industrial application as a central axis, taking into account high solids load conditions and analyzing the toxicity of additives in the fermentation process. This reinforces the practical and integrated nature of the proposal and ensures that the developed strategy is compatible with a complete bioconversion chain. The choice of BSG, an abundant agro-industrial residue that is difficult to utilize due to its high lignin content, reflects a realistic and challenging scenario for biorefinery processes. In this work, we chose to study the effect of the surfactants Tween 80, PEG 6000, PEG 4000, PEG 400, Triton X, and the compounds ferric chloride 3, soy protein, hydrolyzed milk protein (whey), and a cationic polymer on the enzymatic hydrolysis of pretreated BSG. To this end, we aimed to study the behavior of using one or more additives and their interactions on the recovery of glucose obtained after the enzymatic hydrolysis of BSG. Although the effects of additives on the enzymatic hydrolysis of lignocellulosic materials have been extensively studied, to date, no study has been found examining the use of these specific additives in the enzymatic hydrolysis of BSG, nor the effect of using a mixture of these additives using an experimental design based on the response surface methodology (RSM). Therefore, the main objective of this research is to understand the effect of additive mixtures on the enzymatic hydrolysis of BSG pretreated with Ca­(OH)_2_, to maximize the yield of fermentable sugars.

## Materials and Methods

### Enzymes

The commercial complex (CellicCtec2) was used to perform the enzymatic hydrolysis. This complex has its best operational stability in long assays at a temperature of 50 °C, and a pH of 4.8.

### Substrates

The brewer’s spent grain was generously provided by a brewery in the state of Goiás, Brazil. It was collected during the mashing stage, packed in plastic bags, and sustained at 0 °C.

### Additives

The surfactants tested were Tween 80 (NEON, Suzano, SP, Brazil), PEG 6000 (NEON, Suzano, SP, Brazil), PEG 4000 (Synth, Diadema, SP, Brazil), PEG 400 (Synth, Diadema, SP, Brazil), Triton X-100 (NEON, Suzano, SP, Brazil), as well as the compounds ferric chloride 3 (FeCl3·6H_2_O) (NEON, Suzano, SP, Brazil), and the proteins soy and hydrolyzed milk protein (Whey) (Dymatize Enterprises) and also a cationic Polymer (2-propenoic acid, sodium, polymer with 2-propenamide, ceded by a regional sanitation company). While the exact chemical identity and purity are confidential, this polymer is commonly employed in flocculation processes to remove suspended solids. In the present study, it was used as an additive to modulate the hydrophilic–hydrophobic balance during enzymatic hydrolysis of biomass, potentially facilitating lignin removal and enhancing sugar release. All surfactants, compounds and proteins will be called additives for the sake of convenience.

### Sugar Analysis

Glucose determination was carried out using a commercial Glucose Oxidase/Peroxidase Doles (GODPAP) colorimetric kit and high-performance liquid chromatography (HPLC). The method is colorimetric and the indication of glucose concentration in solution is measured in a spectrophotometer (KASUAKI IL–226-NM-BI) at a wavelength of 510 nm (nanometer). Samples were also tested by HPLC for glucose quantification. A Shimadzu chromatography with a Shimpack SCR-102­(H) column and 5 mM perchloric acid aqueous solution as the mobile phase, with a flow rate of 0.6 mL/min and a column oven temperature of 50 °C, was used for analysis.

### Chemical Characterization of Brewer’s Spent Grain

Samples of brewer’s spent grain in natura and pretreated were analyzed to determine moisture, ash, soluble and insoluble lignin. The methodology used for the chemical characterization of BSG before and after pretreatment was determined by the analysis of the products of acid hydrolysis according to Gouveia.^23^ For the determination of moisture, a moisture balance (Bel i-thermo G163L) was used. For ash analysis, 2 g of dry sample and 10 mL of 72% (v/v) sulfuric acid were added to Falcon tubes. The samples were heated to 45 °C for 10 min with stirring in a shaker (Alfa Mare AM800). Samples were autoclaved (autoclave -STERMAX) at 121 °C for 30 min and the solid fraction was separated from the liquid by filtration on filter paper. The solid fraction was used for the determination of ash and insoluble lignin. The liquid fraction of the filtrate was used for analysis in the spectrophotometer to quantify the soluble lignin.

### Alkaline Pretreatment of Brewer’s Spent Grain

Pretreatment with calcium hydroxide was carried out based on an earlier study and a more recent one that repeated the experiment to expand the available data in the literature.^24,25^

To carry out the alkaline pretreatment, initially a lime solution in the proportion of 0.1 g lime/1 g of BSG was added to a flask and a ratio of 9 mL of water/1 g of dry biomass was also used. After homogenizing the mixture, the system was taken to an autoclave (STERMAX), at operating conditions of 120 °C for 1 h. At the end of the reaction, after the mixture reached room temperature, the solid fraction was separated by filtration and washed with water and buffer solution (pH 4.0). Since the treated biomass has a high pH value (>10), which can cause inhibition of enzymatic activity, reducing the pH until reaching a pH close to neutral is essential for efficient enzymatic hydrolysis. The dried samples were stored under refrigeration for later analysis.

### Enzymatic Hydrolysis of Brewer’s Spent Grain

The Enzymatic hydrolysis was carried out in 100 mL Erlenmeyer flasks, using the enzyme (CellicCtec2), for temperature values at 50 °C, and 0.05 M citrate buffer pH 4.8, with an agitation of 200 rpm. The enzyme loading was 15 FPU (Filter Paper Unit) /g dry biomass and with a solid loading of 10% (w/v). The hydrolysis time varied from 8 to 24 h, with sampling times of 0, 0.5, 1, 2, 4, 8, and 24 h. For hydrolysis carried out with additives, a prior adsorption step was performed, in which the additives were added using citrate buffer (0.05 M, pH 4.8) containing the biomass. The mixture was subjected to mechanical agitation at 200 rpm in a reactor (IKA RW20) for 2 h and stored at 4 °C for 12 h. After this time, the biomass was filtered and subjected to enzymatic hydrolysis using the same citrate buffer. To evaluate the results of the enzymatic hydrolysis of BSG, glucose recovery was presented. Glucose recovery is given by the ratio between the experimental glucose obtained and the theoretical glucose contained in the pretreated biomass. The theoretical glucose of the pretreated biomass is calculated by multiplying the mass of cellulose by 1.11, a factor that corrects the difference in mass between anhydrous cellulose and monomeric glucose formed by hydrolysis.

### Data Analysis

All statistical analyses presented in the following sections were performed using the STATISTICA software package (StatSoft, Inc.) and PAST (PAleontological STatistics). Analysis of variance (ANOVA) was employed to assess the presence of statistically significant differences among the various surfactant groups examined, as well as among samples containing additives at 5%, 10%, and 15% solid loadings. When significant effects were detected, Tukey’s post hoc test was applied at a 5% significance level to determine specific group differences. Furthermore, the response surface methodology (RSM) was utilized to model and optimize the effects of the additives PEG 6000, Tween 80, and Polymer on the enzymatic hydrolysis of pretreated brewer’s spent grain, using the STATISTICA software (StatSoft, Inc.).

### Study of the Effect of the Use of Additives on the Enzymatic Hydrolysis of Pretreated Brewer’s Spent Grain

The selection of additives used to verify their effect on the enzymatic hydrolysis of biomass was based on previous research in which surfactants were tested. The investigated additives include**:** Tween 80 (0.005:0.05 g/g Dry Mass (DM));^26^ PEG 6000 (0.005:0.05 g/g DM);^27,28^ PEG 4000 (0.03:0.05 g/g DM);^29,30^ PEG 400 (0.01:0.05 g/g DM);^31,32^ Triton X (0.0005:0.05 g/g DM);^33^ Ferric chloride 3 (0.01 g/g DM);^34,35^ soy protein (0.04 g/g DM);^28^ hydrolyzed milk protein (Whey) (0.025 g/g DM)^35^ and cationic Polymer­(0.0005:0.05 g/g DM).^36^

In this research it was possible to identify that the use of additives at a concentration of 0.05 g of additive/g of dry biomass resulted in the best glucose yield responses in the enzymatic hydrolysis of the pretreated material. Initial enzymatic biomass hydrolysis tests were carried out to evaluate the effect of the nine additives used in this study, in addition to an experiment without additives (control) for comparison.

The data obtained were analyzed using the PAST software through analysis of variance (ANOVA), followed by Tukey’s multiple comparison test at a 5% significance level (*p* < 0.05), to determine the presence of statistically significant differences among the treatments.

### Study of the Effects of the Mixture of the Additives PEG 6000, Tween 80 and Cationic Polymer on the Enzymatic Hydrolysis of Brewer’s Spent Grain

To study the effects of the combination of additives that present better results in the conversion of cellulose into glucose, a central composite rotational design (RCCD) with two levels and three variables (2^3^) was carried out, in addition to three replicates in the central point and six experiments in the axial points (α = 1.68179), totalling 17 runs. The analysis of the design was carried out using the STATISTICA program (Statsoft). The additives studied that showed the best results are PEG 6000, Tween 80 and Cationic Polymer, and the levels of the concentration of these variables were chosen according to the literature and previous tests. The three variables were studied in four main levels: low (−1 = 0.005 g/g), high (+1 = 0.025g/g), −α (0 g/g) and +α (0.0317 g/g). The levels can be seen in Table 1.

**Table 1:** Values of the Levels of the Independent Variables Used in the First RCCD Experiment 2^3^

1° experiment RCCD 2^3^					
varieties	levels				
	+1	–1	0	+α	–α
additives	0.025	0.005	0.015	0.0317	0

The experiments described in the experimental design were carried out in a shaker while maintaining all the temperature, pH, and stirring parameters specified in Enzymatic hydrolysis of brewer’s spent grain section.

The results obtained in this first experimental design suggested the choice of PEG 6000 additives and the Polymer that showed better glucose yields from pretreated BSG (see Results and Discussions section). Therefore, a new RCCD2^2^ was carried out, aiming to maximize the yield of fermentable sugars (variable response).

For study of these two additives, a second central composite design was carried out, with two levels and two variables (2^2^), as well as three repetitions and two axil points (α = 1.41421), the levels can be seen in Table 2.

**Table 2:** Values of the Levels of the Independent Variables Used in the Second RCCD Experiment 2^2^

2° experiment RCCD 2^2^					
varieties	levels				
	+1	–1	0	+α	–α
additives	0.03	0.005	0.018	0.035678	0.000322

The mathematical model found after the factorial planning was validated using concentration values of the additives PEG 6000 and cationic polymer within the range studied in the planning. Table 3 shows the values used in the experiments carried out to validate the model.

**Table 3:** Values of the Variables Used in the Model Validation of the Second Design

experiments	PEG 6000 (g/g)	polymer (g/g)
1	0.024	0.023
2	0.023	0.024
3	0.021	0.018

### PEG 6000 and Polymer Cationic Toxicity Test

Two fermentation experiments were carried out to evaluate the possible toxicity of the concentrations, obtained in the first experimental design of the additives PEG 6000 and Polymer on the fermentative process of the hydrolysates. In one of the experiments, a mixture of 0.1 g/L PEG 6000 and 0.005 g/L Polymer was added to the culture medium (YPD Medium: Yeast extract, Peptone and Dextrose), while in the other the mixture added to the medium was 0.1 g/L Polymer and 0.005 g/L PEG 6000. The fermentation experiments were carried out using the yeast *Sacharomyces cerevisiae* (0.5 g/L), the additives and the YPD medium (10 g/L Yeast Extract, 20 g/L Peptone and 20 g/L Glucose) in an incubator chamber with orbital shaking at 30 °C for 24 h.

### Effect on the Use of Additives in Different Loads of Solids in the Hydrolysis of Pretreated Brewer’s Spent Grain

To evaluate the effect of using additives at different substrate concentrations, enzymatic hydrolysis experiments were carried out in triplicate under the following operating conditions: temperature of 50 °C, pH 4.8, enzyme load 15 FPU/g, concentration of 0.018 g/g PEG 6000 and 0.018 g/g Cationic Polymer, these additives concentration being which allowed to obtain the best glucose yield. The solid loads tested were 5%, 10% and 15% (w/v) and enzymatic hydrolysis was evaluated in the reaction time of 24 h.

## Results and Discussion

### Chemical Characterization of Raw and Pretreated Brewer’s Spent Grain

In this study, the chemical composition of BSG was analyzed, both in its natural state and after pretreatment with calcium hydroxide, as illustrated in Table 4.

**Table 4:** Chemical Characterization of BSG before and after Alkaline Pretreatment

component	biomass in nature (%)	pretreated biomass (%)
cellulose	16.5	30
insoluble lignin	28	24.79
soluble lignin	3.2	3
ash	1.48	1.88

The results showed that the alkaline treatment was quite effective: the cellulose content in the biomass increased significantly, from 16.5% to 30%, representing an 80% increase. This cellulose enrichment made the material much more suitable for enzymatic hydrolysis. The increased glucose release confirms that the cellulose fraction became more concentrated, as the pretreatment removed noncellulosic components such as lignin, hemicelluloses, and other soluble compounds.

Lignin removal, on the other hand, was modest: the insoluble lignin content fell by only 3.2%, from 28% to 24.79%. This low removal is due to the formation of a complex between calcium and lignin, which hinders its solubilization.^18,37,38^ Previous work indicates that, depending on the substrate and reagent, some phases of lignin degradation may be absent, and that alkaline pretreatments with short residence times do not present a residual delignification phase accompanied by carbohydrate degradation.^39,40^ This modest level of delignification is consistent with results reported for other lignocellulosic materials, such as rice straw and corn straw.^41,42^ Although less efficient than sodium hydroxide, calcium hydroxide was chosen because it is cheaper, easily recovered, and better aligned with green technology principles.^43,44^

Although delignification was minimal, pretreatment with Ca­(OH)_2_ increased cellulose accessibility and biomass porosity. Maintaining a high lignin content was an intentional strategy: we wanted to investigate how additives perform precisely in more challenging scenarios, in which lignin serves as a barrier to enzyme action. The main objective was to test the efficiency of additives in overcoming this barrier and improving enzymatic hydrolysis even in the presence of high lignin levels.

Hemicellulose and residual protein were not quantified in this study. Nevertheless, alkaline pretreatment with Ca­(OH)_2_ at high pH, 120 °C, and in the presence of Ca^2+^, promotes strong denaturation and solubilization of proteins by disrupting hydrogen bonds, ionic interactions, and disulfide bridges, in addition to deamidation and Maillard-type reactions, resulting in irreversible loss of the native conformation.^45–47^ Thus, many proteins are removed in the washing steps, and the performance of enzymatic hydrolysis is mainly associated with interactions with lignin and the effects of additives, as reported for alkaline-pretreated lignocellulosic substrates.

### Enzymatic Hydrolysis of Pretreated Brewer’s Spent Grain and the Effect of Using Additives

Given the scarcity of studies on the use of additives to improve the enzymatic hydrolysis of BSG, our research investigated the effectiveness of nine different substances. We tested surfactants (Tween 80, PEG 6000, PEG 4000, PEG 400, Triton X), compounds (ferric chloride), proteins (soy protein, whey), and a cationic polymer, all aimed at increasing glucose recovery. The glucose yield profiles due to cellulose conversion in enzymatic hydrolysis for the use of each additive are shown in Figure 1.

**Figure 1:**
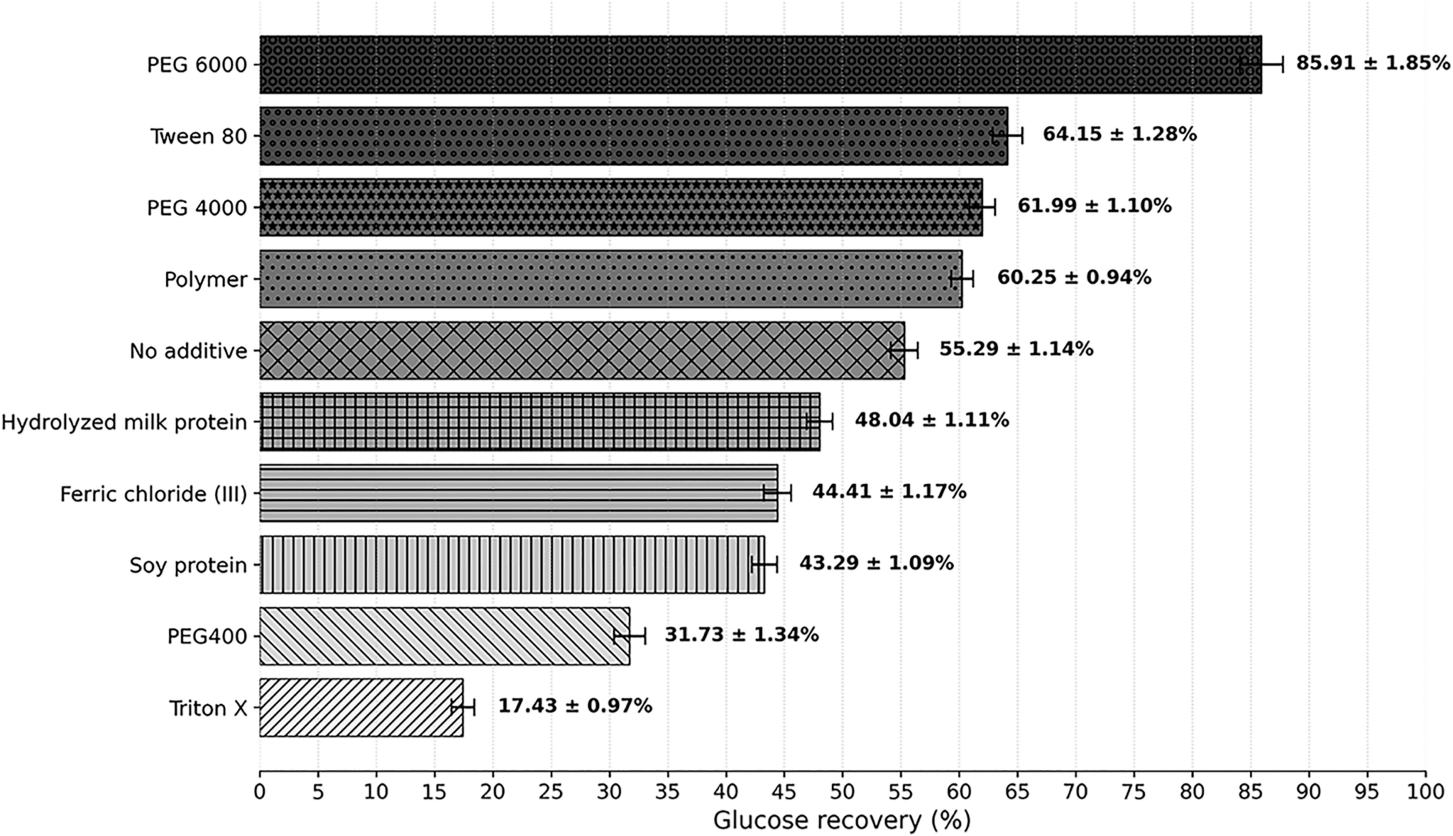
Glucose yield after 8 h of enzymatic hydrolysis of BSG pretreated with the use of different additives.

In the control experiments (without additives), glucose conversion was 55.29%. Lower results were observed with ferric chloride (44.11%), soy protein (43.29%), whey (48.04%), PEG 400 (31.73%), and Triton X (17.43%). In contrast, significant gains were achieved with PEG 6000 (85.91%), PEG 4000 (61.99%), Tween 80 (64.15%), and cationic polymer (60.52%).

Surfactants act through multiple mechanisms: they facilitate enzymatic release of the substrate, stabilize enzymes, and block their adsorption to lignin through adsorption to the hydrophobic lignocellulosic surface.^19^ Effectiveness varies depending on the complex interactions between lignin, enzyme, and additive.^48^ BSG has a high lignin content (28% m/m),^49^ higher than wheat straw (21.2%),^50^ rice straw (12.4%),^51^ sugar cane bagasse (21.35%),^52^ corn straw (19.5%),^42^ and palm kernel (24.29%).^34^ Structurally, its lignin presents a predominance of guaiacylic units and p-hydroxycinnamates, a characteristic that directly influences the action of additives.^53,54^

The effectiveness of additives is highly dependent on the type of biomass and lignin properties, explaining the inconsistent results in the literature.^55^ Soy protein and whey, which improved hydrolysis in sugar cane bagasse, were ineffective here.^35,56^ The same occurred with Triton X and PEG 400, which were successful on other substrates^34,57,58^ but not in this study. Contradictory results with Triton X were observed on other biomasses.^35,59^

The benefits with PEG 6000/PEG 4000 are attributed to its hydrophilicity and greater number of ethylene oxide units, which reduce interfacial tension and form regions of lower polarity.^60,61^ This mechanism allows PEG 6000 to adsorb to the hydrophobic groups of lignin, while its hydrophilic portion protects the enzymes, redirecting them to cellulose and hemicellulose. The cationic polymer also showed promising results, consistent with the literature.^36^ Studies with other biomasses confirm the potential of these additives: Tween 80 in Jabon wood pulp, PEG 6000 in sugar cane bagasse and poplar sawdust, and cationic polymers in waste paper.^62–64^ Interestingly, in palm and wheat, the effects of Tween 80 and PEG 6000 were similar, while the Fe^3+^/Tween 80 combination showed a synergistic effect in wheat straw.^28,65^

The addition of Polyacrylamide (C-PAM) promoted an increase of 8.8%, while the addition of Poly­(ethylenimine) (PEI) promoted an increase of 15.4%, and finally, the use of cationic starch (CA) promoted an increase of 2.6%, these cationic polymers were systematically compared with nonionic additives to identify the most effective formulations. As illustrated in Figure 1, the additives PEG 6000, Tween 80, PEG 4000, and the cationic polymer yielded the highest average glucose recoveries during the enzymatic hydrolysis of pretreated brewer’s spent grain. To investigate the statistical significance of these differences, an analysis of variance (ANOVA) carried out, followed by Tukey’s multiple comparisons test, adopting a significance level of 5%. The ANOVA revealed statistically significant differences between the mean values of the additives (*p*-value = 4.5 × 10^–23^), with PEG 6000 standing out as the highest among them and differing from the others. On the other hand, the yields obtained with PEG4000 and the cationic polymer did not differ statistically from each other, being considered equivalent in terms of efficacy.

As illustrated in Figure 1, the treatment means showed wide variation in glucose yield values, ranging from 17.43% to 85.91%, demonstrating substantial differences between treatments. This disparity suggests the presence of significant systematic effects attributable to the different surfactants tested. Furthermore, the standard deviations within each treatment were low, resulting in coefficients of variation (CV) below 5%, demonstrating high experimental precision and homogeneity between replicates. The combination of the wide variation between treatments (between-group effect) and the low variability within groups (experimental error) resulted in a high *F*-value in the analysis of variance, accompanied by an extremely small *p*-value (*p* = 4.5 × 10^–23^). These values reinforce the reliability of the results obtained regarding the influence of surfactants on glucose yield.

Therefore, for the sequential analyses, it was decided to use the additives PEG 6000, Tween 80, and the cationic polymer. The exclusion of PEG 4000 is justified by the significant structural and functional similarity between PEG 4000 and PEG 6000, both belonging to the nonionic polyether family. The inclusion of the cationic polymer broadens the scope of the investigation, allowing the evaluation of synergistic effects between additives with different modes of action and their impact on the enzymatic hydrolysis of complex lignocellulosic matrices.

### Optimizing the Use of Additives PEG 6000, Tween 80, and Polymer in the Enzymatic Hydrolysis of Pretreated Brewer’s Spent Grain

Tests with additives in the enzymatic hydrolysis of BSG (Figure 1) demonstrated that PEG 6000, Tween 80, and cationic polymer, at concentrations below 0.05 g/g, significantly increased glucose yield. To systematically evaluate this effect, a rotational central composite design (RCCD 2^3^) was implemented, whose experimental matrix, including coded variables and hydrolysis results is presented in Table 5.

**Table 5:** Experimental Matrix (RCCD 2^3^) with the Uncoded Variables and the Results of the Response Variable Conversion of Cellulose into Glucose Resulting from the Enzymatic Hydrolysis at 8 h

experiment	PEG 6000 (g/g)	Tween 80 (g/g)	polymer (g/g)	glucose recovery within 8 h (%)
**1**	(−1)0.005	(−1)0.005	(−1)0.005	60.2
**2**	(−1)0.005	(−1)0.005	(+1)0.025	78.1
**3**	(−1)0.005	(+1)0.025	(−1)0.005	73.5
**4**	(−1)0.005	(+1)0.025	(+1)0.025	69.3
**5**	(+1)0.025	(−1)0.005	(−1)0.005	80.1
**6**	(+1)0.025	(−1)0.005	(+1)0.025	59.1
**7**	(+1)0.025	(+1)0.025	(−1)0.005	75.5
**8**	(+1)0.025	(+1)0.025	(+1)0.025	69.2
**9**	(−α)0	(0)0.015	(0)0.015	68.4
**10**	(+α)0.0317	(0)0.015	(0)0.015	66.2
**11**	(0)0.015	(−α)0	(0)0.015	59
**12**	(0)0.015	(+α)0.0317	(0)0.015	53.4
**13**	(0)0.015	(0)0.015	(−α)0	64.1
**14**	(0)0.015	(0)0.015	(+α)0.0317	59.3
**15**	(0)0.015	(0)0.015	(0)0.015	59.6
**16**	(0)0.015	(0)0,015	(0)0.015	47
**17**	(0)0.015	(0)0.015	(0)0.015	53.9

Glucose yields ranged from 47% to 80.1% over the studied range. Experiment 5, with the highest concentration of PEG 6000, achieved the highest yield (80.1%), while Experiment 2, with the highest cationic polymer content, achieved 78.1%. In contrast, Tween 80 showed limited contribution: its variation from −1 to +1 (experiments 1 and 3) resulted in only a 22% increase in yield, lower than the 33% and 30% observed for PEG 6000 and Cationic Polymer, respectively. These results suggest significant synergy between PEG and Polymer, but little influence of Tween 80 on this substrate. Table 6 illustrates the analysis of variance for the terms of the model reduced to the 10% significance level.

**Table 6:** Analysis of Variance for the Terms of the Reduced Model for the RCCD 2^3^ ^a^

factor	SS	DF	MS	*F*	*P*
PEG 6000(Q)	0,043137	1	0,043137	8,816151	0,010864
Polymer (Q)	0,019075	1	0,019075	3,898472	0,069963
Interaction	0,021012	1	0,021012	4,294409	0,058686
Error	0,063609	13	0,004893		
Total SS	0,136446	16			

a SS: sum of squares, DF: degrees of freedom, MS: mean square, *F*: *f* factor and *P*: *p* value.

Although the coefficient of determination (*R* ^2^) was modest (54%), the model allowed us to identify regions of interest and guide subsequent optimization steps. Residue analysis (Figure 2) showed more pronounced variability at low predicted values, indicating some heterogeneity in the data. A reduced regression model (eq 1) was fitted based on statistical significance (Table 6).

1 $$\mathrm{glucose}\left(\%\right)=0.562+0.057\;\mathrm{PE}G^{2}+0.042\;\mathrm{polyme}r^{2}-0.05\;\mathrm{PEG}\times \mathrm{polymer}$$

**Figure 2:**
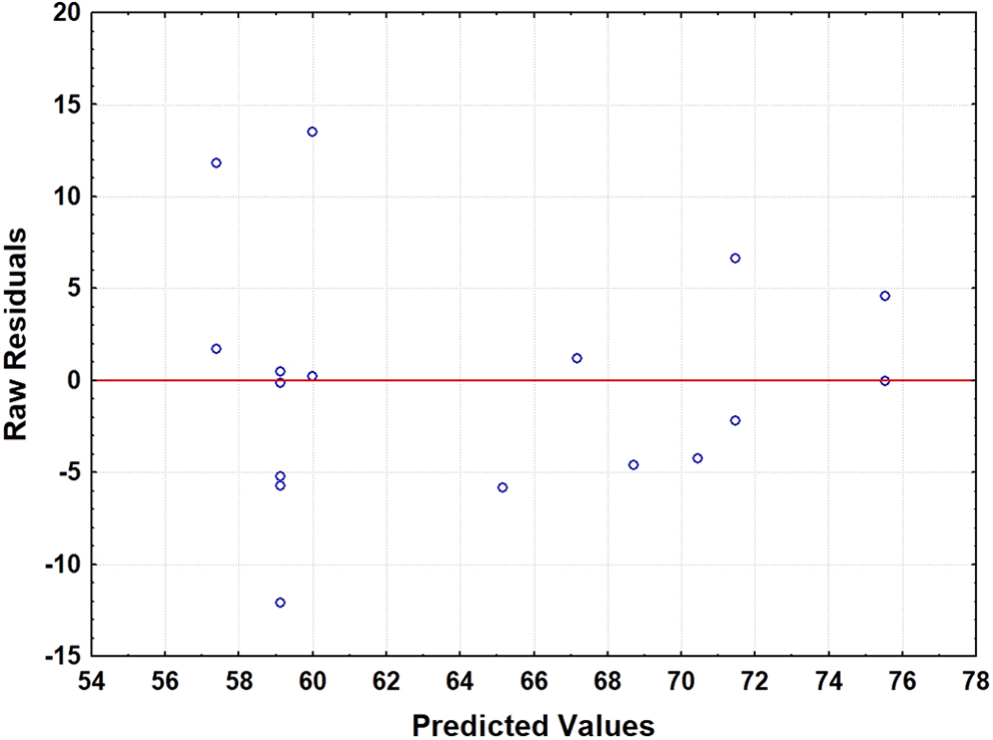
Raw residuals versus predicted values plot for the RCCD2^3^.

The response surfaces (Figure 3) confirmed the absence of a maximum point within the tested range, but highlighted that Tween 80 had no significant effect on glucose recovery (Figure 3a,3b), contrary to what was observed with other biomasses.^62^ On the other hand, PEG 6000 exhibited a consistent positive effect (Figure 3c), possibly due to its preferential adsorption on hydrophobic groups of lignin, freeing the enzymes to act on the polysaccharides.^61^

**Figure 3:**
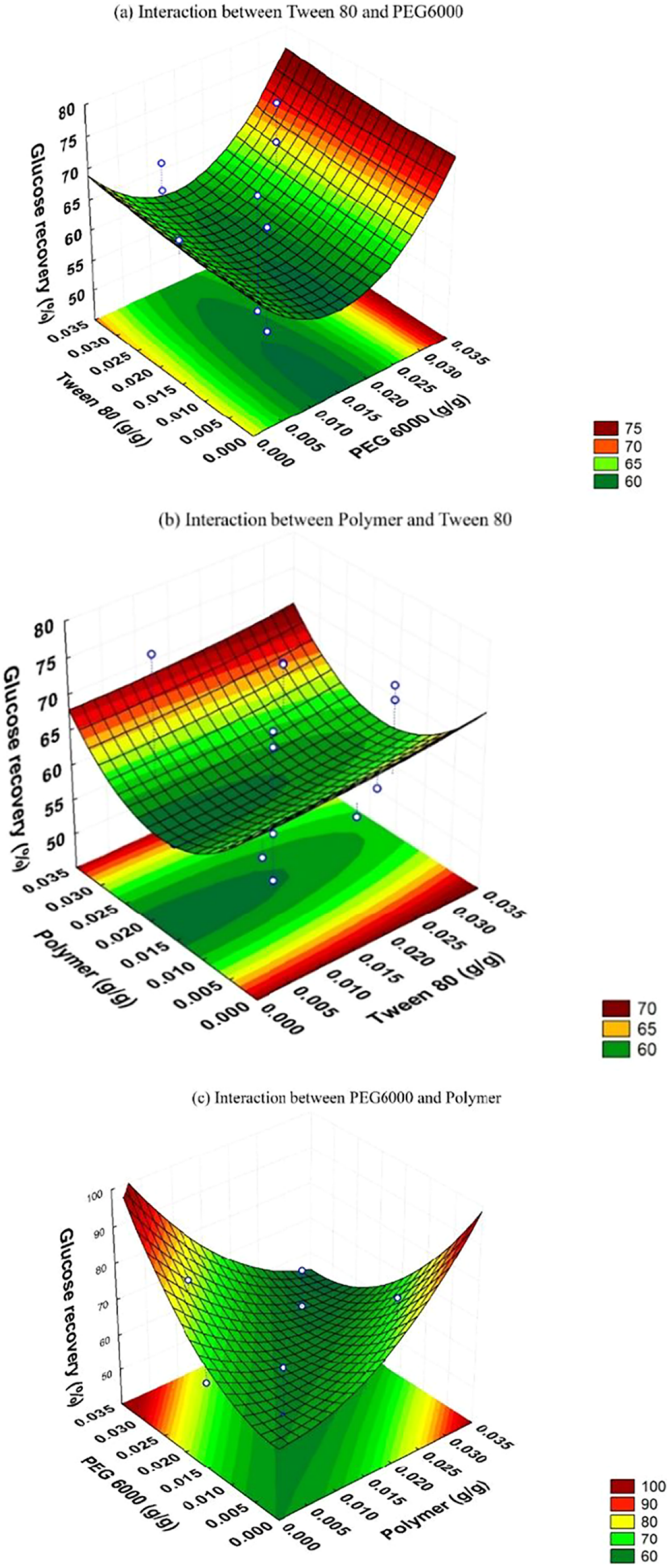
Response surface, obtained from RCCD 2^3^, for the conversion of cellulose to glucose resulting from enzymatic hydrolysis as a function of the interaction between additives: (a) Tween 80 and PEG 6000; (b) Tween 80 and Polymer and (c) PEG 6000 and Polymer.

Compared to previous studies, such as the one that obtained 40.3% glucose recovery in Jabon wood pulp using Tween 80.^62^ The results reported here reinforce that the effectiveness of additives is strongly dependent on the substrate. The structural and compositional uniqueness of BSG therefore requires specific optimization strategies.

Given these insights, a new experimental design was conducted, focusing exclusively on PEG 6000 and cationic polymer, with the aim of identifying optimal conditions and maximizing hydrolytic yield.

Complementary to these findings, recent investigations sought to elucidate how PEG interacts with lignin surfaces, thus explaining its role in reducing nonproductive enzymatic adsorption and increasing hydrolysis efficiency. Thus, understanding the mechanisms of interaction between poly­(ethylene glycol) (PEG) and lignin is essential to explain its contribution to enhancing enzymatic hydrolysis. The study by Han et al.^66^ demonstrated that PEG reduces lignin hydrophobicity, decreasing nonproductive cellulase adsorption and increasing glucose yield in model systems containing cellulose (Avicel) and acid-insoluble lignin (AICS-lignin).

The proposed mechanism indicates that PEG acts as a physical barrier, blocking adsorption sites and simultaneously altering the hydrophobicity and hydrogen bonding capacity of lignin. To confirm these effects, the authors carried out analyses of adsorption rate, hydrophobicity, glucose yield, and zeta potential, using the TGLC fusion protein as a cellulase surrogate, which allowed for the precise characterization of enzyme–substrate interactions.

Furthermore, Li et al.^67^ demonstrated that the hydrophilic fraction of PEG favors the formation of a hydrated layer on the lignin surface, acting as a steric barrier to enzyme access and increasing its hydrophilicity. This effect promotes a “physical loosening” of the lignocellulosic matrix, improving cellulose’s accessibility to enzymes.

Finally, microscopic analyses revealed more diffuse and less compact microstructures in treated samples, while FTIR spectra confirmed the absence of chemical changes in the cellulose. Thus, it is concluded that PEG acts primarily as a physical modifier of the biomass, increasing enzyme accessibility and, consequently, the overall hydrolysis efficiency.

Response surface analysis (Figure 3c) revealed two distinct regions that favor glucose yield: one with a high concentration of PEG 6000 (0.025 g/g) and a low cationic polymer (0.005 g/g), resulting in 80.1% recovery; and another with a low PEG (0.005 g/g) and a high polymer (0.025 g/g), reaching 78.1%. These results confirm the compatibility between the experimental data and the regions of maximum efficiency identified by the Response Surface Methodology (RSM).

For the two selected additives a toxicity test was carried out in a fermentation process using the hydrolysates obtained from biomass. The choice to evaluate only two combinations of additives in the fermentation stage was based on using the combinations that produced the highest yields of reducing sugars in the enzymatic hydrolysis stage. Fermentation experiments were carried out using the yeast *S. cerevisiae* at a concentration of 0.5 g cells/L at 30 °C for 24 h. In one experiment, a mixture of 0.1 g/L of PEG 6000 and 0.005 g/L of Polymer was added to the culture medium (YPD Medium), while in the other experiment the mixture added to the medium was 0.1 g/L of Polymer and 0.005g/L of PEG 6000.

Toxicity tests with *S. cerevisiae* demonstrated that high concentrations of the cationic polymer inhibited yeast growth, while PEG 6000 showed noninhibitory behavior consistent with previous reports that attribute protective properties to this surfactant against toxic compounds.^68^ This differential effect guided the selection of conditions for subsequent optimization. Thus, based on the preliminary results obtained in fermentations conducted with the addition of additives and the results obtained in the first RCCD (experimental design 1) to maximize glucose recovery, it was decided to evaluate the impact of the additives PEG 6000 and polymer on the enzymatic hydrolysis of BSG. Consequently, the concentrations were adjusted, and new g/g ratio values were evaluated (Table 7).

**Table 7:** Experimental Matrix of RCCD 2^2^ Showing the Uncoded Variables and the Results of the Response Variable Glucose Recovery in Enzymatic Hydrolysis over 24 h in a Shaker

experiment	PEG 6000 (g/g)	polymer (g/g)	glucose recovery within 24 h (%)
**1**	(−1)0.005	(−1)0.005	72.6
**2**	(−1)0.005	(+1)0.03	76.5
**3**	(+1)0.03	(−1)0.005	84.5
**4**	(+1)0.03	(+1)0.03	66.0
**5**	(−α)0.0003	(0)0.018	70.0
**6**	(+α)0.035	(0)0.018	73.2
**7**	(0)0.018	(−α)0.0003	76.2
**8**	(0)0.018	(+α)0.035	76.6
**9**	(0)0.018	(0)0.018	87.7
**10**	(0)0.018	(0)0.018	87.9
**11**	(0)0.018	(0)0.018	86.1

The new Rotational Central Composite Design (RCCD) 2^2^ (α = 1.41) was implemented with two variables at two levels, along with three central replicates and four axial points, totaling 11 experiments.

The experimental design was carried out considering a significance level of 90% (*p*-value <0.1). The maximum glucose recovery response obtained in the hydrolysis was 87.9% in the second RCCD (experimental design 2) (Table 7), being higher than the 80.1% obtained in the initial RCCD experimental design (Table 5).

The results of the new RCCD 2^2^ design, which focused exclusively on the interactions between PEG 6000 and the cationic polymer, showed that glucose recovery ranged from 66% to 87.9%, with the best performances (>80%) occurring under the central conditions (0.018 g/g of both additives), which obtained high process reproducibility and the highest glucose yields. These experiments share a common feature: both present PEG 6000 and polymer at the central concentration (0.018 g/g), indicating that there is a positive interaction between these factors to maximize glucose recovery.

In contrast, experiment 4, with maximum concentrations of both additives (0.03 g/g), resulted in a drastic drop in yield (66%). This suggests that there may be a limit to the amount of additives that can improve the performance of the hydrolysis process. These results suggest that glucose recovery does not increase indefinitely with increasing additive concentrations, but rather that there is an optimal loading limit of these compounds to improve process yield. This result indicates that there appears to be a maximum loading of these additives with which it is possible to obtain a higher glucose yield using the brewer’s spent grain.

Based on the data in Table 7, a statistical analysis of the effects and interactions was carried out in the central rotational composite design. Nonsignificant coefficients (*p*-value >0.1) were excluded, resulting in a reduced multiple regression model. This model demonstrated a strong correlation (*R* ^2^ = 94%) between the concentration of additives (PEG 6000 and cationic polymer) and glucose recovery in the enzymatic hydrolysis of BSG, indicating a good fit to the experimental data.

The derived statistical model (eq 2) showed a good fit (*R* ^2^ = 94%)

2 $$\mathrm{glucose}\left(\%\right)=87.22-7.59\;\mathrm{PE}G^{2}-5.19\;\mathrm{polyme}r^{2}-5.59\;\mathrm{PEG}\times \mathrm{polymer}$$

where, glucose (%), corresponds to the recovery of glucose in the enzymatic hydrolysis of the pretreated brewer’s spent grain. Table 8 illustrates the analysis of variance for the significant terms of the model reduced to the 10% significance level and Figure 4 presents the plot of raw residuals versus predicted values for the variable glucose yield (%), allowing the assessment of randomness and the absence of patterns in the model errors.

**Figure 4:**
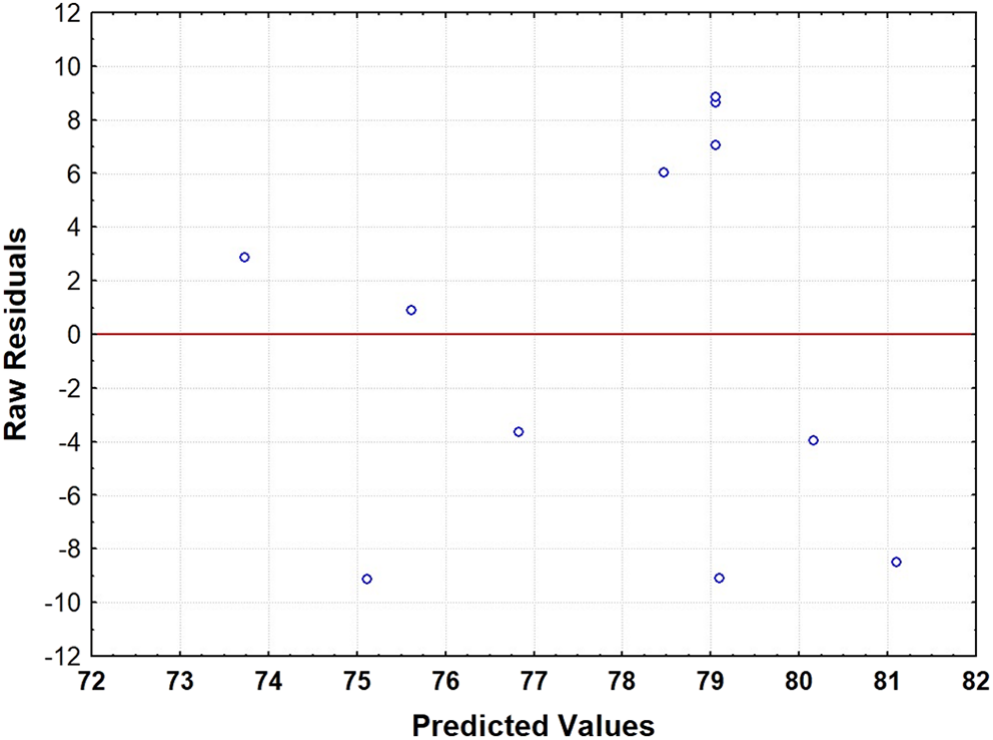
Raw residuals versus predicted values plot.

**Table 8:** Analysis of Variance for the Terms of the Reduced Model for the RCCD2^2^ ^a^

factor	SS	DF	MS	*F*	*P*
PEG 6000(Q)	325.4592	1	325.4592	36.42424	0.000524
Polymer (Q)	152.2075	1	152.2075	17.03452	0.004419
Interaction	125.4400	1	125.4400	14.03880	0.007198
Error	62.5467	7	8.9352		
Total SS	567.5655	10			

a SS: sum of squares, DF: degrees of freedom, MS: mean square, *F*: *f* factor and *P*: *p* value.

Figure 4 presents the plot of raw residuals versus predicted values for the variable glucose yield (%), allowing the assessment of randomness and the absence of patterns in the model errors.

The analysis of variance (ANOVA), summarized in Table 8, confirmed the statistical significance of the quadratic terms and the interaction between the additives (*p* < 0.01), validating the structure of the proposed model. Additionally, the residual analysis, illustrated in Figure 4, did not reveal the presence of systematic patterns, with the residuals exhibiting a symmetrical distribution around the zero-reference line. This behavior corroborates the randomness of the errors and the adequacy of the model to the experimental data.

The derived response surface (Figure 5) identified a region of maximum efficiency, delimited by the range of 0.015–0.025 g/g for both additives, with a predicted optimum point at 0.019 g/g of PEG 6000 and 0.014 g/g of Cationic Polymer, corresponding to a maximum glucose recovery yield of 87.2%. The agreement between the predicted and experimental results reinforces the robustness of the model and its applicability in process optimization.

**Figure 5:**
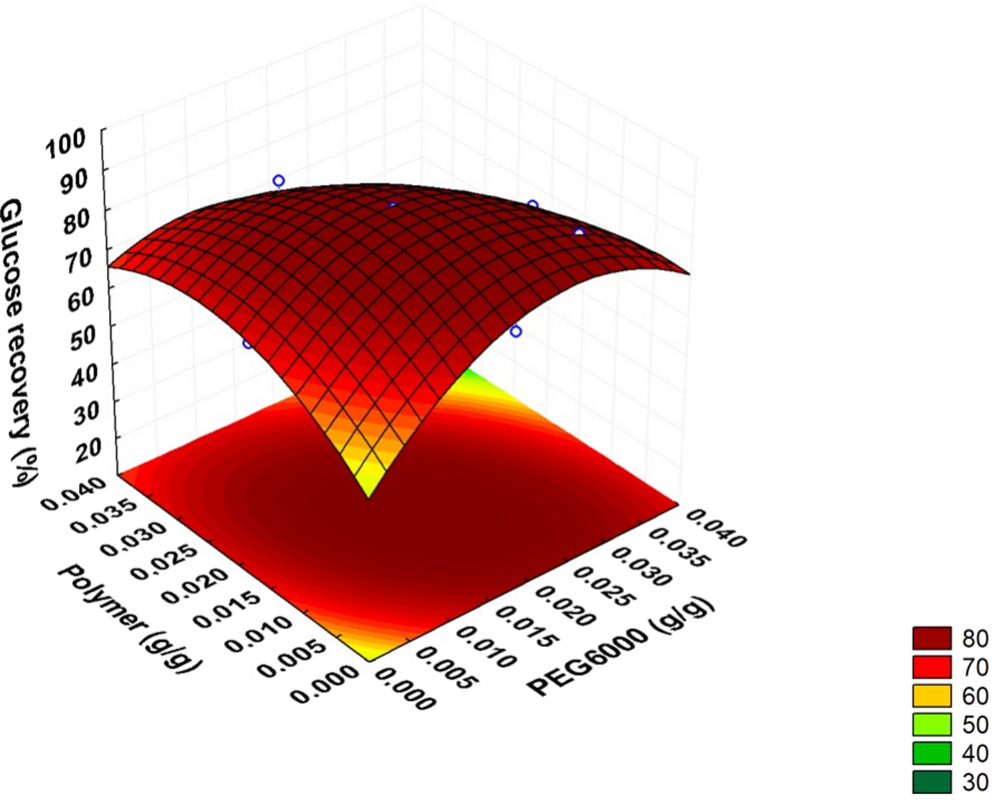
Response surface obtained from RCCD 2^2^ for the conversion of cellulose to glucose by enzymatic hydrolysis as a function of PEG 6000 additives and low Cationic Polymer.

From the statistical model, it is possible to demonstrate how the effect of the interaction between the concentrations of the additives PEG 6000 and Cationic Polymer significantly affects glucose recovery. The analysis of the response surface, as illustrated in Figure 5, indicates a clear interaction between PEG 6000 and the polymer in the response. It can be observed that glucose recovery is maximized in a specific region of the experimental space, where both variables are close to the central level or slightly shifted to positive values. Thus, it is suggested that PEG 6000 and the polymer act to stabilize the enzyme structure. PEG 6000, due to its hydrophilic nature, can increase enzyme solubility, while the cationic polymer tends to modulate the interactions between glucose and the reaction medium, favoring its recovery.

The derived response surface (Figure 5) stipulates a region of maximum efficiency, delimited by the range of 0.015–0.025 g/g for both additives, with a predicted optimum at 0.019 g/g of PEG 6000 and 0.014 g/g of Cationic Polymer, corresponding to a maximum glucose recovery yield of 87.2%. The surface also shows that small deviations toward higher PEG 6000 concentrations can positively contribute to recovery, but the synergy between the two components appears to play a key role in achieving yields above 80%. Finally, the agreement between the predicted and experimental results reinforces the robustness of the model and its applicability in process optimization.

This interaction between the additives suggests the existence of an ideal balance point, at which glucose recovery is maximized without compromising process efficiency. Ultimately, for industrial applications, this balance needs to be precisely tuned to ensure high efficiency and minimize reagent waste, thus ensuring the robustness of the system and its large-scale predictions.

The experiments were conducted using the maximum concentrations obtained as specified in Table 9 to verify the validity of the results of the statistical model (eq 2).

**Table 9:** Percent Error between Hydrolysis Conversion Responses Obtained by the Model and Experimentally

experiments	PEG 6000 (g/g)	polymer (g/g)	(%) experimental conversion	(%) model conversion	error (%)
**1**	0.024	0.023	87.7	87.2	0.57
**2**	0.023	0.024	86.7	88.39	1.9
**3**	0.021	0.018	82.6	86.52	4.7

Experimental validation under near-optimal conditions (Table 9) showed an error of less than 5% between predicted and observed values, confirming the robustness of the model. These results clearly demonstrate that the synergy between PEG and polymer is maximized within a narrow range of concentrations, beyond which antagonistic effects may occur. This nonlinear relationship is critical for industrial applications, where the balance between efficiency and cost must be rigorously optimized.

### Effect of Substrate Concentration [S]

To evaluate the effect of additives under conditions of higher biomass concentration, enzymatic hydrolysis of BSG was carried out using an increase in solids loading (5%, 10%, and 15% w/v) and the previously experimentally determined optimal concentrations (0.018 g PEG 6000/g biomass and 0.018 g Cationic Polymer/g biomass). The results demonstrated that increasing solids loading significantly impacted glucose yield (Figure 6). In the hydrolysis process in which the additives were used at concentrations that generated maximum glucose recoveries for solids contents of 5% and 10%, glucose recovery proved efficient, with results close to the maximum response point of 94.54% and 85.50%, respectively. The use of these additives allowed a gain of up to 30% compared to the control system. Furthermore, the use of the additive had a positive effect on enzymatic hydrolysis at solids content of 15%, resulting in a 15.7% increase in glucose recovery compared to the control system, with a maximum recovery rate of 69.05%. This positive effect demonstrates that the use of additives also improves process yield even with higher solids loads, making the lignocellulosic conversion process more economically viable.^69,70^

**Figure 6:**
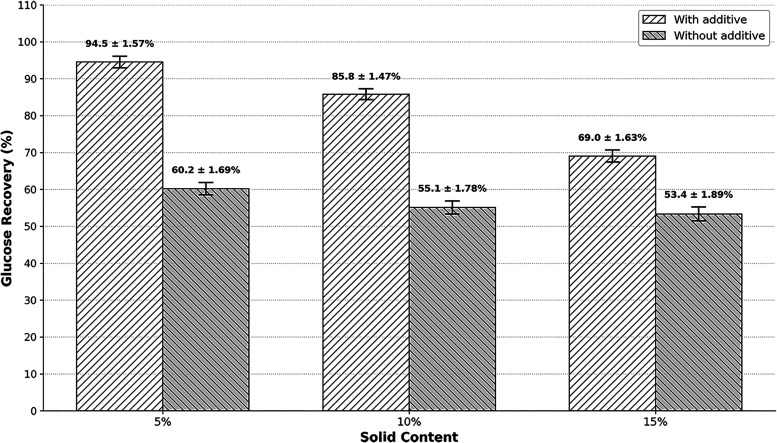
Glucose yield in the hydrolysis of brewer’s spent grain for different solids contents of 5%, 10% and 15%, using a mixture of the additives PEG 6000 (0.018 g/g) and Cationic Polymer (0.018 g/g).

The behavior shown is expected, as the increase in solids load in the hydrolysis process presents greater challenges, one of them being the lack of available water in the reactor, which is essential for efficient hydrolysis, mainly due to phenomena such as mass transfer and lubrication of the reaction medium. Specifically, water provides a means to solubilize and assist in the mass transfer of the products, while also reducing the viscosity of the reaction medium, thus facilitating the mixing process and material handling.^69,70^

Using the software PAST, analysis of variance (ANOVA) was carried out on samples containing additives with 5%, 10%, and 15% solids, using a significance level of 5%. The results indicated statistically significant differences among all evaluated conditions. Tukey’s test, also conducted at a 5% significance level (*p*-value = 2.89 × 10^–6^), revealed that all samples were statistically different, with the 5% solids sample showing the highest glucose recovery mean among the treatments analyzed.

Despite the significant gains obtained with the use of additives, it is crucial to recognize that the reduction in yield at higher solids concentrations is associated with challenges such as mass transfer limitations, increased viscosity, product inhibition (glucose and cellobiose), and nonproductive enzyme adsorption.^71^ Previous studies report similar behavior in other biomasses, with a decrease in yield above 15% solids.^72,73^ In the present study, a consistent decline was observed above 5% solids, in line with the specialized literature.

In the specific case of this residue, the high lignin concentration could result in low hydrolytic efficiency; however, our results demonstrate that the appropriate use of PEG 6000 and cationic polymer additives promotes selective adsorption on the hydrophobic sites of lignin, reducing nonproductive enzyme adsorption and improving the production of fermentable sugars.

It is crucial to emphasize that processing with high solids loads is industrially relevant. The optimized use of these additives allowed significant glucose recoveries even at 15% solids, making the process more economically viable. The effectiveness of the additives, however, was shown to be dependent on the type of biomass and its specific characteristics, particularly the lignin content and nature.^17^ In BSG, the interaction between additives and lignocellulosic components proved to be essential for releasing enzymes to act on polysaccharides.

This study reinforces, therefore, that additive selection and dosage must be carefully optimized for each substrate, considering its compositional and operational characteristics, in order to maximize the efficiency of the enzymatic hydrolysis process.

## Conclusion

Alkaline pretreatment with calcium hydroxide increased the cellulose content of BSG from 16.5% to 30% and, combined with the use of additives, significantly improved enzymatic hydrolysis. Among the additives tested, PEG 6000 and the cationic polymer showed a synergistic effect, achieving glucose yields of up to 87.7% under optimized conditions. Application at different solids loadings (up to 15%) confirmed their effectiveness, although performance decreased at higher levels. In enzymatic hydrolysis, this study suggests that the appropriate use of PEG 6000 additives and cationic polymer promotes the adsorption process of the additive with lignin, thus improving the production of fermentable sugars. These results highlight the potential of the combined use of PEG 6000 and a cationic polymer to overcome lignin barriers and intensify enzymatic conversion, but they also highlight challenges for industrial scale-up, such as costs, sustainability, and toxicity. Future research should focus on more severe solids loading conditions, the environmental and economic impacts of additives, and the utilization of residual lignin.

## References

[1] Chaudhary G., Chaudhary N., Saini S., Gupta Y., Vivekanand V., Panghal A. (2024). Assessment of Pretreatment Strategies for Valorization
of Lignocellulosic Biomass: Path Forwarding towards Lignocellulosic
Biorefinery. Waste Biomass Valorization.

[2] Ibarra-Gonzalez P., Christensen L. P., Rong B.-G. (2022). A Critical Review of Separation Technologies
in Lignocellulosic Biomass Conversion to Liquid Transportation Fuels
Production Processes. Chem. Eng. Commun..

[3] Puligundla P., Mok C. (2021). Recent Advances in
Biotechnological Valorization of Brewers’
Spent Grain. Food Sci. Biotechnol..

[4] López-Linares J. C., García-Cubero M. T., Lucas S., González-Benito G., Coca M. (2019). Microwave
Assisted Hydrothermal as Greener Pretreatment of Brewer’s
Spent Grains for Biobutanol Production. Chem.
Eng. J..

[5] Rathour R. K., Behl M., Dhashmana K., Sakhuja D., Ghai H., Sharma N., Meena K. R., Bhatt A. K., Bhatia R. K. (2023). Non-food
crops derived lignocellulose biorefinery for sustainable production
of biomaterials, biochemicals and bioenergy: a review on trends and
techniques. Ind. Crop. Prod..

[6] Sajib M., Falck P., Sardari R. R. R., Mathew S., Grey C., Karlsson E. N., Adlercreutz P. (2018). Valorization
of Brewer’s Spent
Grain to Prebiotic Oligosaccharide: Production, Xylanase Catalyzed
Hydrolysis, In-Vitro Evaluation with Probiotic Strains and in a Batch
Human Fecal Fermentation Model. J. Biotechnol..

[7] da
Silva E. G., Borges A. S., Maione N. R., Castiglioni G. L., Suarez C. A. G., Montano I. D. C. (2020). Fermentation of Hemicellulose Liquor
from Brewer’s Spent Grain Using *Scheffersomyces
Stipitis* and *Pachysolen Tannophilus* for Production of 2G Ethanol and Xylitol. Biofuels Bioprod. Bioref..

[8] Rommi K., Niemi P., Kemppainen K., Kruus K. (2018). Impact of Thermochemical
Pre-Treatment and Carbohydrate and Protein Hydrolyzing Enzyme Treatment
on Fractionation of Protein and Lignin from Brewer’s Spent
Grain. J. Cereal Sci..

[9] Haldar D., Purkait M. K. (2021). A Review on the
Environment-Friendly Emerging Techniques
for Pretreatment of Lignocellulosic Biomass: Mechanistic Insight and
Advancements. Chemosphere.

[10] Wagle A., Angove M. J., Mahara A., Wagle A., Mainali B., Martins M., Goldbeck R., Raj Paudel S. (2022). Multi-Stage
Pre-Treatment of Lignocellulosic Biomass for Multi-Product Biorefinery:
A Review. SETA.

[11] Shen J., Zheng Q., Zhang R., Chen C., Liu G. (2019). Co-Pretreatment
of Wheat Straw by Potassium Hydroxide and Calcium Hydroxide: Methane
Production, Economics, and Energy Potential Analysis. J. Environ. Manage..

[12] Chetrariu A., Dabija A. (2020). Pre-Treatments Used for the Recovery
of Brewer’s
Spent Grain—A Minireview. J. Agroaliment.
Processes Technol..

[13] Madadi M., Song G., Gupta V. K., Aghbashloh M., Sun C., Sun F., Tabatabaei M. (2023). Non-Catalytic Proteins as Promising
Detoxifiers in Lignocellulosic Biomass Pretreatment: Unveiling the
Mechanism for Enhanced Enzymatic Hydrolysis. Green Chem..

[14] Kumar A., Minuye N., Ayele Y. B., Yadav M. (2018). A Review of Factors
Affecting Enzymatic Hydrolysis of Pretreated Lignocellulosic Biomass. Res. J. Chem. Environ..

[15] Ravindran R., Sarangapani C., Jaiswal S., Lu P., Cullen P. J., Bourke P., Jaiswal A. K. (2019). Improving Enzymatic Hydrolysis of
Brewer Spent Grain with Nonthermal Plasma. Bioresour.
Technol..

[16] Santos F. A., de H., Colodette J. L., Fernandes S. A., Rezende S. T. (2011). Potential of Sugarcane
Straw for Ethanol Production. Quim. Nova.

[17] AL-Azkawi, A. ; AL-Battashi, H. ; Sivakumar, N. Nonionic Surfactants for Enhancement of Lignocellulose Enzymatic Hydrolysis. In Recent Developments in Bioenergy Research; Elsevier, 2020; pp 225–246.

[18] Jiang D., Ge X., Zhang Q., Zhou X., Zhou C., Keener H. M., Li Y. (2017). Comparison of Sodium Hydroxide and Calcium Hydroxide Pretreatments
of Giant Reed for Enhanced Enzymatic Digestibility and Methane Production. Bioresour. Technol..

[19] Wang W., Zhuang X., Tan X., Wang Q., Chen X., Yu Q., Qi W., Wang Z., Yuan Z. (2018). Dual Effect of Nonionic
Surfactants on Improving the Enzymatic Hydrolysis of Lignocellulose. Energy Fuels.

[20] Huang C., Zhao X., Zheng Y., Lin W., Lai C., Yong Q., Ragauskas A. J., Meng X. (2022). Revealing the Mechanism
of Surfactant-Promoted Enzymatic Hydrolysis of Dilute Acid Pretreated
Bamboo. Bioresour. Technol..

[21] Brondi M. G., Vasconcellos V. M., Giordano R. C., Farinas C. S. (2019). Alternative Low-Cost
Additives to Improve the Saccharification of Lignocellulosic Biomass. Appl. Biochem. Biotechnol..

[22] Pallapolu V. R., Shi S., Kang L., Kothari U., Li J. (2020). Boost Effect of Water-Soluble
Polymers on Enzymatic Hydrolysis of Lignocellulosic Biomass. Ind. Eng. Chem. Res..

[23] Gouveia E. R., Nascimento R. T., Souto-Maiorana M., Rocha G. J. M. (2009). Validation of
Methodology for the Chemical Characterization of Sugar Cane Bagasse. Quim. Nova..

[24] Chang V. S., Burr B., Holtzapple M. T. (1997). Lime pretreatment
of switchgrass. Appl. Biochem. Biotechnol..

[25] Rabelo S. C., Filho R. M., Costa A. C. (2009). Lime Pretreatment
of Sugarcane Bagasse
for Bioethanol Production. Appl. Biochem. Biotechnol..

[26] Sánchez-Muñoz S., Balbino T. R., de Oliveira F., Rocha T. M., Barbosa F. G., Vélez-Mercado M. I., Marcelino P. R. F., Antunes F. A. F., Moraes E. J. C., dos
Santos J. C., da Silva S. S. (2022). Surfactants, Biosurfactants, and
Non-Catalytic Proteins as Key Molecules to Enhance Enzymatic Hydrolysis
of Lignocellulosic Biomass. Molecules.

[27] Nasirpour N., Mousavi S. M., Shojaosadati S. A. (2014). A Novel
Surfactant-Assisted Ionic
Liquid Pretreatment of Sugarcane Bagasse for Enhanced Enzymatic Hydrolysis. Bioresour. Technol..

[28] Zhou Y., Yang J., Luo C., Yang B., Liu C., Xu B. (2019). Effect of Metal Ions
and Surfactants on the Enzymatic Hydrolysis
of Pretreated Lignocellulose. Bioresources.

[29] Ge X., Sun Z., Xin D., Zhang J. (2014). Enhanced Xylanase Performance in
the Hydrolysis of Lignocellulosic Materials by Surfactants and Non-Catalytic
Protein. Appl. Biochem. Biotechnol..

[30] Li Y., Ge X., Sun Z., Zhang J. (2015). Effect of Additives on Adsorption
and Desorption Behavior of Xylanase on Acid-Insoluble Lignin from
Corn Stover and Wheat Straw. Bioresour. Technol..

[31] Hsieh C.-W., Cannella D., Jørgensen H., Felby C., Thygesen L. G. (2015). Cellobiohydrolase
and Endoglucanase Respond Differently to Surfactants during the Hydrolysis
of Cellulose. Biotechnol. Biofuels Bioprod..

[32] Amândio M. S.
T., Rocha J. M. S., Xavier A. M. R. B. (2023). Enzymatic Hydrolysis Strategies for
Cellulosic Sugars Production to Obtain Bioethanol from Eucalyptus
Globulus Bark. Fermentation.

[33] Pino M. S., Michelin M., Rodríguez-Jasso R. M., Oliva-Taravilla A., Teixeira J. A., Ruiz H. A. (2021). Hot Compressed Water
Pretreatment
and Surfactant Effect on Enzymatic Hydrolysis Using Agave Bagasse. Energies.

[34] Bukhari N. A., Loh S. K., Bakar N. A., Jahim M. D. J. (2018). Enhanced Sugar
Recovery from Oil Palm Trunk Biomass by Repeated Enzymatic Hydrolysis
with Surfactant Addition. Malays. Appl. Biol..

[35] Zhou Y., Chen H., Qi F., Zhao X., Liu D. (2015). Non-ionic
surfactants do not consistently improve the enzymatic hydrolysis of
pure cellulose. Bioresour. Technol..

[36] Florencio C., Badino A. C., Farinas C. S. (2016). Soybean
Protein as a Cost-Effective
Lignin-Blocking Additive for the Saccharification of Sugarcane Bagasse. Bioresour. Technol..

[37] Yu H., Xu Y., Ni Y., Wu Q., Liu S., Li L., Yu S., Ji Z. (2018). Enhanced Enzymatic
Hydrolysis of Cellulose from Waste
Paper Fibers by Cationic Polymers Addition. Carbohydr. Polym..

[38] Rodrigues C. I. S., Jackson J. J., Montross M. D. (2016). A Molar
Basis Comparison of Calcium
Hydroxide, Sodium Hydroxide, and Potassium Hydroxide on the Pretreatment
of Switchgrass and Miscanthus under High Solids Conditions. Ind. Crops Prod..

[39] Kaur, M. Effect of Hydrogen Peroxide & Calcium Hydroxide Pretreatment of Wheat Straw on Biogas Production Mater. Today: Proc. 2024 10.1016/j.matpr.2024.05.092.

[40] Fuentes L. L. G., Rabelo S. C., Filho R. M., Costa A. C. (2011). Kinetics of Lime
Pretreatment of Sugarcane Bagasse to Enhance Enzymatic Hydrolysis. Appl. Biochem. Biotechnol..

[41] Jančíková V., Jablonský M. (2024). Exploiting Deep Eutectic Solvent-like Mixtures for
Fractionation Biomass, and the Mechanism Removal of Lignin: A Review. Sustainability.

[42] Du J., Qian Y., Xi Y., Lü X. (2019). Hydrothermal
and Alkaline Thermal Pretreatment at Mild Temperature in Solid State
for Physicochemical Properties and Biogas Production from Anaerobic
Digestion of Rice Straw. Renewable Energy.

[43] Chen X., Liu S., Zhai R., Yuan X., Yu Y., Shen G., Wang Z., Yu J., Jin M. (2022). Lime Pretreatment of
Pelleted Corn Stover Boosts Ethanol Titers and Yields without Water
Washing or Detoxifying Pretreated Biomass. Renewable
Energy.

[44] Kumar S., Gandhi P., Yadav M., Paritosh K., Pareek N., Vivekanand V. (2019). Weak Alkaline Treatment of Wheat and Pearl Millet Straw
for Enhanced Biogas Production and Its Economic Analysis. Renewable Energy.

[45] Dissanayake M., Ramchandran L., Donkor O. N., Vasiljevic T. (2013). Denaturation
of whey proteins as a function of heat, pH and protein concentration. Int. Dairy J..

[46] Paker I., Jaczynski J., Matak K. E. (2017). Calcium hydroxide
as a processing
base in alkali-aided pH-shift protein recovery process. J. Sci. Food Agric..

[47] Peng Y., Dewi D. P. A. P., Kyriakopoulou K., van der Goot A. J. (2020). Effect
of calcium hydroxide and fractionation process on the functional properties
of soy protein concentrate. IFSET.

[48] Imman S., Khongchamnan P., Wanmolee W., Laosiripojana N., Kreetachat T., Sakulthaew C., Chokejaroenrat C., Suriyachai N. (2021). Fractionation and Characterization of Lignin from Sugarcane
Bagasse Using a Sulfuric Acid Catalyzed Solvothermal Process. RSC Adv..

[49] Noonari A. A., Shah A. R., Mirjat N. H., Anh T. (2023). Various Pretreatments
of Canola Straw with Hydrogen Peroxide, Calcium Hydroxide, Silica,
and Pleurotus Ostreatus to Improve Methane Yield through Anaerobic
Co-Digestion. Biomass Convers. Biorefin..

[50] Lobo G. C., Almeida J. B., Rodrigues D. S., Suarez C. A. G., Cavalcanti I. D. M. (2023). Mass
Balance and Water Demand in the Alkaline Pretreatment Process of the
Main Brewing Residue. Cad. UniFOA.

[51] Lauberte L., Telysheva G., Cravotto G., Andersone A., Janceva S., Dizhbite T., Arshanitsa A., Jurkjane V., Vevere L., Grillo G., Gaudino E. C., Tabasso S. (2021). Lignin – Derived Antioxidants
as Value-Added
Products Obtained under Cavitation Treatments of the Wheat Straw Processing
for Sugar Production. J. Cleaner Prod..

[52] Rosado M. J., Rencoret J., Marques G., Gutiérrez A., del Río J. C. (2021). Structural Characteristics of the
Guaiacyl-Rich Lignins
from Rice (*Oryza Sativa* L.) Husks and
Straw. Front. Plant Sci..

[53] Meneses N. G. T., Martins S., Teixeira J. A., Mussatto S. I. (2013). Influence of Extraction
Solvents on the Recovery of Antioxidant Phenolic Compounds from Brewer’s
Spent Grains. Sep. Purif. Technol..

[54] Rencoret J., Prinsen P., Gutiérrez A., Martínez Á. T., Río J. C. D. (2015). Isolation
and Structural Characterization
of the Milled Wood Lignin, Dioxane Lignin, and Cellulolytic Lignin
Preparations from Brewer’s Spent Grain. J. Agric. Food Chem..

[55] Xu C., Zhang J., Zhang Y., Guo Y., Xu H., Liang C., Wang Z., Xu J. (2019). Lignin Prepared
from
Different Alkaline Pretreated Sugarcane Bagasse and Its Effect on
Enzymatic Hydrolysis. Int. J. Biol. Macromol..

[56] Huang C., Jiang X., Shen X., Hu J., Tang W., Wu X., Ragauskas A., Jameel H., Meng X., Yong Q. (2022). Lignin-Enzyme
Interaction: A Roadblock for Efficient Enzymatic Hydrolysis of Lignocellulosics. Renewable Sustainable Energy Rev..

[57] Xu C., Asraful Alam Md., Wang Z., Chen H., Zhang J., Huang S., Zhuang W., Xu J. (2021). Mechanisms
of Bio-Additives
on Boosting Enzymatic Hydrolysis of Lignocellulosic Biomass. Bioresour. Technol..

[58] Lee S., Akeprathumchai S., Bundidamorn D., Salaipeth L., Poomputsa K., Ratanakhanokchai K., Chang K.-L., Phitsuwan P. (2021). Interplays
of Enzyme, Substrate, and Surfactant on Hydrolysis of Native Lignocellulosic
Biomass. Bioengineered.

[59] Qu X.-S., Hu B.-B., Zhu M.-J. (2017). Enhanced
Saccharification of Cellulose
and Sugarcane Bagasse by Clostridium Thermocellum Cultures with Triton
X-100 and β-Glucosidase/CellicCTec2 Supplementation. RSC Adv..

[60] Nogueira C. d. C., Padilha C. E. A., Filho P. S., Santos E. (2022). Effects of
the Addition of Poly­(ethylene Glycol) and Non-Ionic Surfactants on
Pretreatment, Enzymatic Hydrolysis, and Ethanol Fermentation. Bioenergy Res..

[61] Medeiros J. D., Kanis L. A. (2010). Evaluation of PEG
effects on the extracts obtaining
from Passiflora edulis Sims, Passifloraceae, and Mikania glomerata
Spreng., Asteraceae. Rev. Bras. Farmacogn..

[62] Nababan M. Y. S., Fatriasari W., Wistara N. J. (2022). Response Surface Methodology for
Enzymatic Hydrolysis Optimization of Jabon Alkaline Pulp with Tween
80 Surfactant Addition. Biomass Convers. Biorefin..

[63] Baral P., Jain L., Kurmi A. K., Kumar V., Agrawal D. (2020). Augmented
Hydrolysis of Acid Pretreated Sugarcane Bagasse by PEG 6000 Addition:
A Case Study of Cellic CTec2 with Recycling and Reuse. Bioproc. Biosyst. Eng..

[64] Lai C., Jia Y., Yang C., Chen L., Shi H., Yong Q. (2020). Incorporating
Lignin into Polyethylene Glycol Enhanced Its Performance for Promoting
Enzymatic Hydrolysis of Hardwood. ACS Sustainable
Chem. Eng..

[65] Vergara P., Ladero M., Carbajo J. M., Garcia-Ochoa F., Villar J. C. (2021). Effect of Additives on the Enzymatic Hydrolysis of
Pre-Treated Wheat Straw. Braz. J. Chem. Eng..

[66] Han L., Jiang B., Wang W., Wang G., Tan Y., Niu K., Fang X. (2022). Alleviating
Nonproductive Adsorption of Lignin on CBM
through the Addition of Cationic Additives for Lignocellulosic Hydrolysis. ACS Appl. Bio Mater..

[67] Li H., Wang C., Xiao W., Yang Y., Hu P., Dai Y., Jiang Z. (2019). Dissecting the effect of polyethylene glycol on the
enzymatic hydrolysis of diverse lignocellulose. Int. J. Biol. Macromol..

[68] Liu X., Xu W., Mao L., Zhang C., Yan P., Xu Z., Zhang Z. C. (2016). Lignocellulosic
Ethanol Production by Starch-Base Industrial
Yeast under PEG Detoxification. Sci. Rep..

[69] Modenbach A. A., Nokes S. E. (2013). Enzymatic Hydrolysis
of Biomass at High-Solids Loadings
– A Review. Biomass Bioenergy.

[70] Wang Y., Qiao H., Tao Y., Ma Z., Zheng Z., Ouyang J. (2023). Addressing Two Major Limitations
in High-Solids Enzymatic
Hydrolysis by an Ordered Polyethylene Glycol Pre-Incubated Strategy:
Rheological Properties and Lignin Adsorption for Enzyme. Bioresour. Technol..

[71] Kristensen J. B., Felby C., Jørgensen H. (2009). Yield-Determining Factors in High-Solids
Enzymatic Hydrolysis of Lignocellulose. Biotechnol.
Biofuels..

[72] Olsen S. N., Borch K., Cruys-Bagger N., Westh P. (2014). The Role of Product
Inhibition as a Yield-Determining Factor in Enzymatic High-Solid Hydrolysis
of Pretreated Corn Stover. Appl. Biochem. Biotechnol..

[73] Weiss N. D., Felby C., Thygesen L. G. (2019). Enzymatic
Hydrolysis Is Limited by
Biomass–Water Interactions at High-Solids: Improved Performance
through Substrate Modifications. Biotechnol.
Biofuels.

